# Characterizing stroke-induced changes in the variability of lower limb kinematics using multifractal detrended fluctuation analysis

**DOI:** 10.3389/fneur.2022.893999

**Published:** 2022-08-05

**Authors:** Pan Xu, Hairong Yu, Xiaoyun Wang, Rong Song

**Affiliations:** ^1^Key Laboratory of Sensing Technology and Biomedical Instrument of Guangdong Province, Sun Yat-sen University, Guangzhou, China; ^2^Guangdong Provincial Engineering and Technology Center of Advanced and Portable Medical Devices, Sun Yat-sen University, Guangzhou, China; ^3^Guangdong Work Injury Rehabilitation Center, Guangzhou, China

**Keywords:** adaptation, multifractality, walking, movement variability, correlation

## Abstract

Movement variability reflects the adaptation of the neuromuscular control system to internal or external perturbations, but its relationship to stroke-induced injury is still unclear. In this study, the multifractal detrended fluctuation analysis was used to explore the stroke-induced changes in movement variability by analyzing the joint angles in a treadmill-walking task. Eight healthy subjects and ten patients after stroke participated in the experiment, performing a treadmill-walking task at a comfortable speed. The kinematics data of the lower limbs were collected by the motion-capture system, and two indicators, the degree of multifractality (α) and degree of correlation [*h*(2)], were used to investigate the mechanisms underlying neuromuscular control. The results showed that the knee and ankle joint angles were multifractal and persistent at various scales, and there was a significant difference in the degree of multifractality and the degree of correlation at the knee and ankle joint angles among the three groups, with the values being ranked in the following order: healthy subjects < non-paretic limb < paretic limb. These observations highlighted increased movement variability and multifractal strength in patients after stroke due to neuromotor defects. This study provided evidence that multifractal detrended analysis of the angles of the knee and ankle joints is useful to investigate the changes in movement variability and multifractal after stroke. Further research is needed to verify and promote the clinical applications.

## Introduction

Walking is an essential human activity and requires a complex set of neuromuscular controls to cope with intrinsic and extrinsic perturbations (1). Walking demonstrates a stable, rhythmic, and oscillatory pattern along with highly irregular fluctuations from time to time (2). The gait pattern contains a lot of information about the normal or pathological state of the subject, which is altered by the persistence of neuromotor defects after stroke (3). The most common stroke-induced injury is a slow and inefficient hemiplegic gait, which is thought to be the result of limited muscle control or loss of muscle control or movement function (4). Recent studies showed that the characterization of gait defects in patients after stroke was essential for understanding the neuromuscular control mechanisms and would help in the design of effective gait rehabilitation strategies for patients after stroke (5).

Gait defects including slow walking speeds (6), gait asymmetry (7), and decreased ankle dorsiflexion (8) severely impair the activity of daily living (ADL) of patients after stroke (9), and numerous methods based on the kinematic, kinetic, and electromyography (EMG) signals have been introduced to study them. Padmanabhan et al. observed that, even after the step length symmetry was improved in patients after stroke, the gait kinematics and kinetics remained markedly asymmetric (10). Hong et al. assessed the postural stability of patients after stroke by extracting characteristics from the center-of-pressure trajectories and proposed the mean velocity of the center of pressure as an effective tool for assessing postural balance (11). Li et al. utilized the surface EMG to identify muscle weakness after stroke and found different patterns in the peak distribution of the surface EMG caused by stroke (12).

Movement variability also serves to describe motor defects. It reflects the adaptability of the neuromuscular control system to internal or external disturbances and has therefore attracted widespread attention (13). Many new methods based on dynamical systems theory have been applied to study motor defects, such as entropy and maximum Lyapunov exponents (14, 15). Ao et al. showed that the complexity of upper limb EMG signals, measured by fuzzy-approximate entropy in the elbow sinusoidal trajectory tracking tasks, increased due to stroke- and aging-induced neurological changes (16). Kempski et al. quantified lower extremity joint angles with the Lyapunov exponent (LyE) and found that the paretic side of patients after stroke exhibited higher structure variability than the non-paretic side (17). Although the aforementioned studies provided new perspectives on the system dynamics, they were prone to interference from nonstationarities and noise in the system (2). Detrended fluctuation analysis (DFA) was proposed to effectively detect the dynamic changes in noisy and nonstationary time series (18, 19). However, DFA could not effectively quantify the complex scaling behavior of many signals, such as geophysical signals, EMG signals, and the stride interval (20–25). Therefore, the standard DFA was extended to capture the multifractal scaling features in the nonstationary time series, and this was called multifractal detrended fluctuation analysis (MFDFA) (26). Many studies showed that movement variability presented a multifractal fluctuation pattern, reflecting the strong physiological interaction that occurs across multiple time scales within the neuromuscular control system (27, 28). Restoring healthy levels of multifractality in the movement variability of patients after stroke enabled them to respond more flexibly to irregularities in the natural environment (29). Therefore, MFDFA might provide a unique perspective on the movement variability of the lower extremities as opposed to traditional gait analysis methods.

Although MFDFA had been used to study the movement variability of the stride interval time series after neurodegenerative diseases in previous studies, there were few studies on the changes in movement variability caused by stroke (18). In this study, a hypothesis is put forward that the gait dynamics of patients after stroke might be less stable, and this is demonstrated by the increased movement variability and multifractality of lower limb joint movement. To verify this hypothesis, the MFDFA is performed using the knee and ankle joint angles signals to explore the differences in the movement variability of the lower extremities between healthy subjects and patients after stroke.

## Methods

### Subjects

In this study, a total of 18 subjects were recruited, including 10 patients after stroke (two women and eight men, age 48.3 ± 12.8 years) and eight healthy subjects (three women and five men, age 29 ± 4.94 years) in the control group. For the paretic and non-paretic groups, the inclusion criteria included the following: (1) chronic stroke survivors (more than 6 months after stroke); (2) the first stroke with unilateral hemiparesis lesions; (3) the ability to walk independently and continuously on a treadmill for at least 5 min; and (4) the ability to follow oral instructions and cooperate with experimental procedures. For the control group, the only inclusion criterion was no history of neuromuscular diseases. All subjects were ambulating independently and were not currently receiving physical therapy. This study was conducted under the approval of the Medical Ethics Committee at the Industrial Injury Rehabilitation Hospital of Guangdong. All subjects had signed a consent form before starting the experiment.

### Experimental setup and procedures

Before the experiment, ten 12-mm reflective markers were affixed to the bilateral lower limbs of each subject and placed at the following anatomical reference positions from bottom to top: the second and third metatarsal space, the lateral malleolus, the midleg, the lateral knee, and the mid-thigh (30). Then, the participants were asked to walk on a treadmill (BH, G6425-F3, Spain) for 5 min wearing specific experimental shoes to familiarize themselves with the experiment and to find the most comfortable speed by adjusting the treadmill speed. During the experiment, subjects were asked to walk on a treadmill for 3 min at a comfortable speed, and this activity was repeated 3 times. There was a 5-min rest period between each walking experiment (31). When the subject was walking on the treadmill, their three-dimensional coordinate trajectory was detected by the 6-cameras motion capture system with a 100 Hz sampling rate (OptiTrack, Natural Point, USA). The coordinate trajectories of all markers were recorded using Tracking Tools software (NaturalPoint, USA) and were processed using MATLAB (MathWorks, Natick, USA) to calculate the joint angle time series for further analysis.

### Multifractal detrended fluctuation analysis

The movement variability in knee and ankle joint angles was quantified by MFDFA. The three groups of the joint angle time series were analyzed as follows: (i) dominant limbs in healthy subjects, (ii) paretic limbs, and (iii) non-paretic limbs of the patients after stroke. The specific steps were as follows (21):

Construct the profile:For the time series *x*(*i*), *i* = 1⋯*N*, its average value $x_{ave}=\frac{1}{N}\overset{N}{\underset{i=1}{\sum }}x\left(i\right)$ was subtracted and the profile *Y*(*i*) was constructed by the cumulative sum of these differences:


(1) $$\begin{array}{r} Y\left(i\right)=\overset{N}{\underset{i=1}{\sum }}\left[x\left(i\right)-x_{ave}\right] \end{array}$$

Divide *Y*(*i*) into *N*_*s*_ = int(*N*/*s*) nonoverlapping segments: Here, *s* is the length of each equal-length segment. However, the length of the time series *N* was scarcely divisible by *s*, and the part that was not divisible might not be calculated. The same calculation process was repeated from the other end. Thus, 2*N*_*s*_ segments were generated in total. Then, the local trend fitting of *l*−order polynomial was performed on each of the generated segments, and the residual variation between the fluctuations between *Y*(*i*) and its *l*− order fit *y*_*v*_(*i*), *i* = 1, ... , *s* was calculated as follows:

For the *v* = 1, ... , *N*_*s*_ segments, we calculated


(2) $$\begin{array}{r} F^{2}\left(s,v\right)\;=\;\frac{1}{s}\overset{s}{\underset{i=1}{\sum }}\{{Y\left[\left(v-1\right)s\;+\;i\right]\;-\;y_{v}\left(i\right)\}}^{2} \end{array}$$

whereas for the *v* = *N*_*s*_+1, ..., 2*N*_*s*_ segments,


(3) $$\begin{array}{r} F^{2}\left(s,v\right)\;=\;\frac{1}{s}\overset{s}{\underset{i=1}{\sum }}{\{Y\left[N-\left(v-N_{s}\right)s\;+\;i\right]\;-\;y_{v}\left(i\right)\}}^{2} \end{array}$$

By calculating the mean of all segments, the *q*_*th*_ order fluctuation function was obtained:


(4) $$\begin{array}{rcl} F_{q}\left(s\right)\; & = & \;{\left\{\frac{1}{2N_{s}}\overset{2N_{s}}{\underset{v=1}{\sum }}[{F^{2}\left(s,v\;\right)]}^{\frac{q}{2}}\right\}}^{\frac{1}{q}} \end{array}$$

(5) $$\begin{array}{rcl} F_{0}\left(s\right)\; & = & \;exp\left\{\frac{1}{4N_{s}}\overset{2N_{s}}{\underset{v=1}{\sum }}ln\left[F^{2}\left(s,v\right)\right]\right\} \end{array}$$

The exponent variable *q* was any real number except zero. If *q* = 0, 1/*q* was infinity, and a different averaging method had to be used, such as logarithmic averaging. To discover the relationship between the generalized fluctuation functions *F*_*q*_(*s*) and the timescale *s* for different *q* values, the above steps were repeated for multiple segments. DFA was a special case of MFDFA when *q* = 2.

Finally, the scaling behavior was determined by plotting *F*_*q*_ vs. *s* on a logarithmic scale and examining the existence of short and long scales. If the fluctuation function could be described as


(6) $$\begin{array}{r} F_{q}\left(s\right)\;\propto \;s^{h\left(q\right)}, \end{array}$$

it meant that the time series had scale characteristics and that the autocorrelation of the time series had no characteristic time scale. Then, we calculated the slope of the fitted straight line of ln *F*_*q*_(*s*) and ln *s* for different values of *q* to obtain the scaling exponent *h*(*q*). In general, the exponent *h*(*q*) was closely related to *q*, and *h*(*q*) was considered to be the generalization of the Hurst exponent.

The generalization of Hurst exponent *h*(*q*) had the following relationship with the classical scaling exponent τ(*q*):


(7) $$\begin{array}{r} \tau \left(q\right)\;=\;qh\left(q\right)\;-\;1 \end{array}$$

The feature of the mono-fractal series was the existence of only a single Hurst exponent *H* with a linear relationship between τ(*q*) and *q*. The multifractal time series would be characterized by multiple Hurst exponents, and τ(*q*) depended nonlinearly on *q*.

The singularity spectrum *f*(α) had the following relationship with *h*(*q*):


(8) $$\begin{array}{rcl} \alpha \; & = & \;h\left(q\right)\;+\;qh^{\prime }\left(q\right) \end{array}$$

(9) $$\begin{array}{rcl} f\left(\alpha \right)\; & = & \;q\left[\alpha \;-\;h\left(q\right)\right]\;+\;1, \end{array}$$

where *f*(α) quantified the dimension of the subset series corresponding to α, which is the singularity strength. A fractal was a repeating pattern that was self-similar across different scales; multifractality referred to patterns that are repeated in multiple ways. Thus, the multifractal spectrum could provide a lot of information about the behavior of fractals in the time series (29). To verify the power law behavior of the fluctuations, the time series were processed by the Fourier spectral analysis first (24, 25, 32). Then, the degree of multifractality was demonstrated by the width of the multifractal spectrum Δα = α_*max*_ − α_*min*_. However, for a nonstationary and random walk-like structure time series, values of the degree of correlation *h*(2) might be above 1. A nonstationary random walk was obtained by integrating stationary noise. Thus, the result *h*(2) reduced by 1 to obtain the eigenvalues for quantifying the persistent and anti-persistent values of the nonstationary random walk time series (33). The value of *h*(2) also revealed the characteristics of long-scale correlation in the time series (21). The origin of multifractality could be determined by processing the time series random shuffling. There were two common sources of multifractals: (i) the extensive probability density function and (ii) the existence of many different long-scale correlation fluctuations in the time series (34). By random shuffling, the time series values would be randomly arranged, and the correlations in the original sequence would be destroyed. Therefore, if the multifractality was the result of the long-scale correlations, then the processed series exhibited a non-fractal scaling. When the origin of multifractality was a broad probability density, the reliance of *h*(*q*) was unchanged, which was unaffected during the reorganization process. If these two multifractals simultaneously appeared, the processed series would exhibit a decrease in the degree of multifractality.

Statistical differences in the values of Δα and *h*(2) for each scaling region between subjects with paretic limbs and healthy subjects, and between subjects with non-paretic limbs and healthy subjects were assessed using an independent *t*-test. Paired *t*-tests were applied between the results of Δα and *h*(2) in those with paretic and those with non-paretic limbs to investigate whether there were statistical differences between the two conditions. For all statistical analyses, the *p*-value of 0.05 significance level was set. The SPSS 24.0 software package was used for all statistical calculations (SPSS Inc., Chicago, USA).

## Result

### Scaling exponents

**Figure 1:**
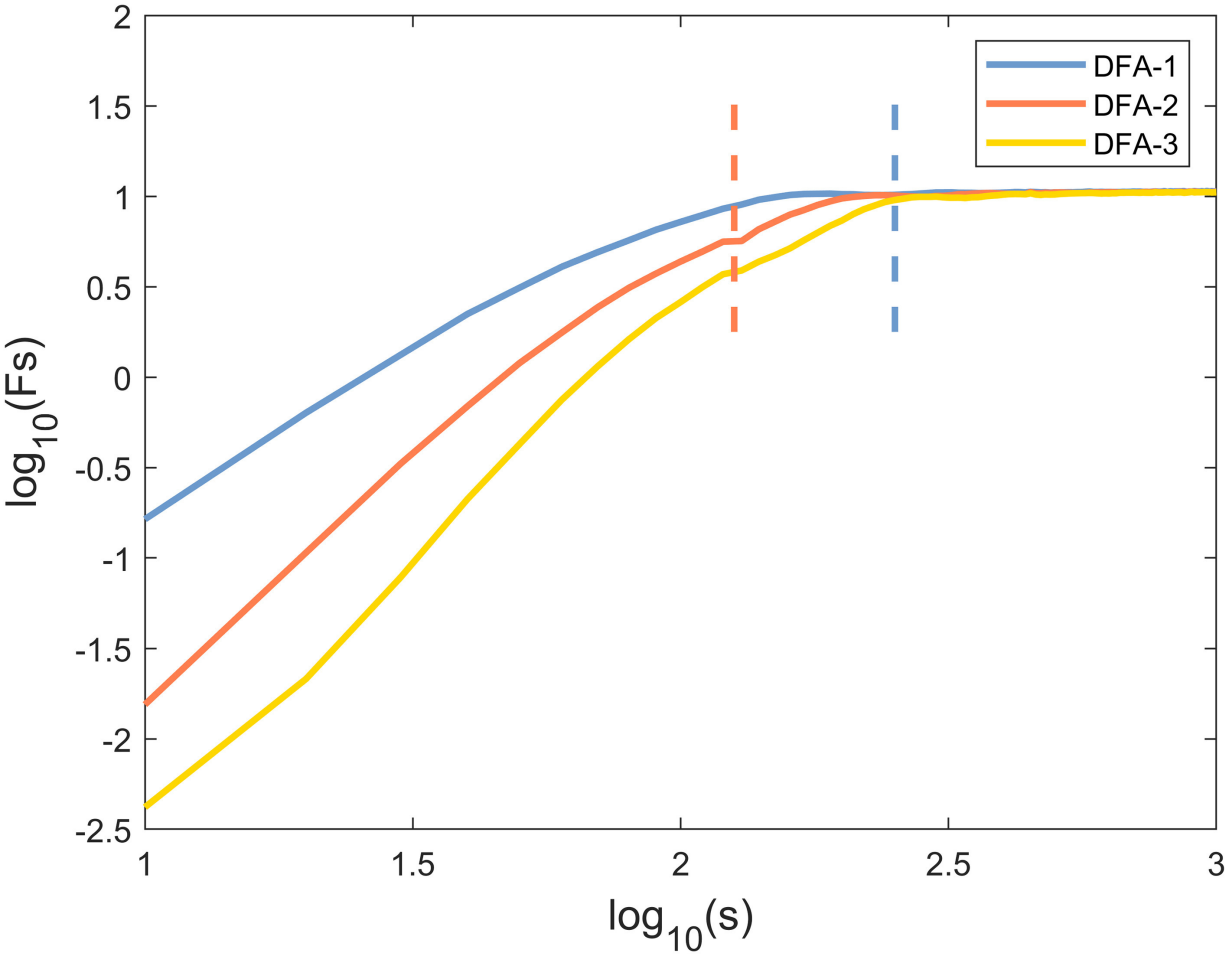
A typical scaling pattern as observed for the knee angle time series of one healthy subject.

**Figure 2:**
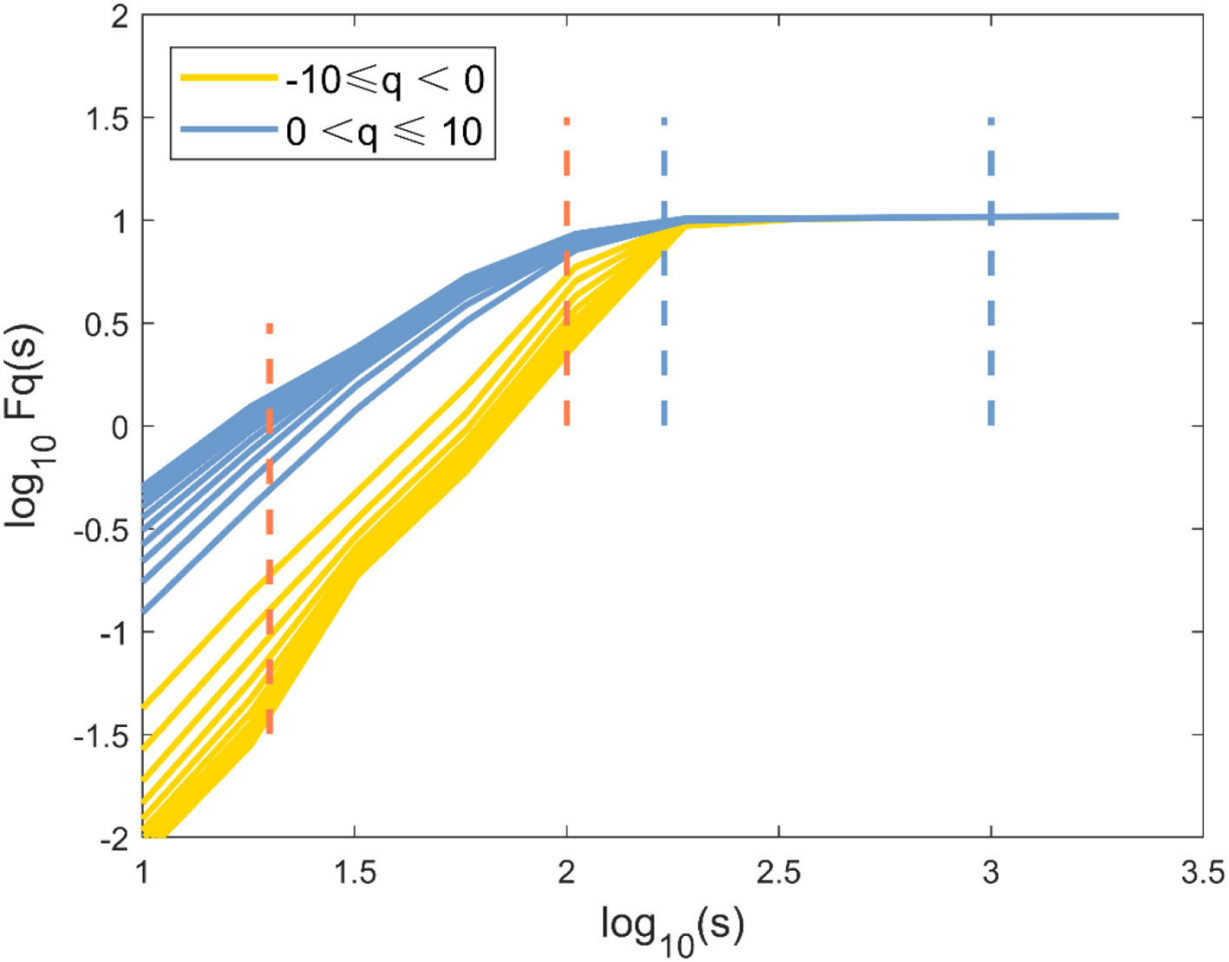
The fluctuation function *logF*_*q*_(*s*) vs. log10(*s*) with −10 ≤ *q* ≤ 10 and its behavior over a range of *s* for knee angle time series of one healthy subject by MFDFA.

The representative examples of the variation of the fluctuation function F with scale s are shown in Figure 1. First, the third-order polynomials (MFDFA1-3) were used in the detrending procedure. F decreased with the increase of the fitting order *l* because the increase of the order would result in a smaller residual. Then, values of *h*(*q*) for −10 ≤ *q* ≤ 10 were displayed to reveal the effect of different magnitudes of joint angles anomalies on a behavior scale. In Figure 2, the fluctuation function *F*_*q*_ of the knee angles' time series of a healthy subject and its behavior with the change of *s* were similar to *F*_2_(*s*) in Figure 1. By performing linear fitting in two independent regions, the characteristics of multifractal scales on the short and long scales were characterized. The vertical red and blue dashed lines represent the boundaries of the short-scale and long-scale regions, respectively. Specifically, the short-scale region started after the DFA transient artifact with the smallest *s* values corresponded to 20 samples and this ended with 100 samples. The long-scale region started with 170 samples and usually ended with *N*/8, and the specific figure corresponded to 1,000 samples. In some joint angles, the phenomenon of crossover was less obvious in the scaling description, and the slopes *h*(*q*) were also estimated over the all-scale region. Furthermore, the all-scale region started the same way as the short-scale region, commencing with 20 samples and usually ending with 1,000 samples. This included the change in slope at the crossover, where the change in slope increased as *q* decreased. By performing linear fitting in the three-scale regions, the characteristics of multifractal scales on the short, long, and all scales were characterized. The vertical red and blue dashed lines are the boundaries of the short-scale and long-scale. The different scale ranges for each participant were the same in this study. The representative fluctuation behavior started with a linear segment with a slope >1, and the slope range was usually between 1.36 and 1.75.

**Figure 3:**
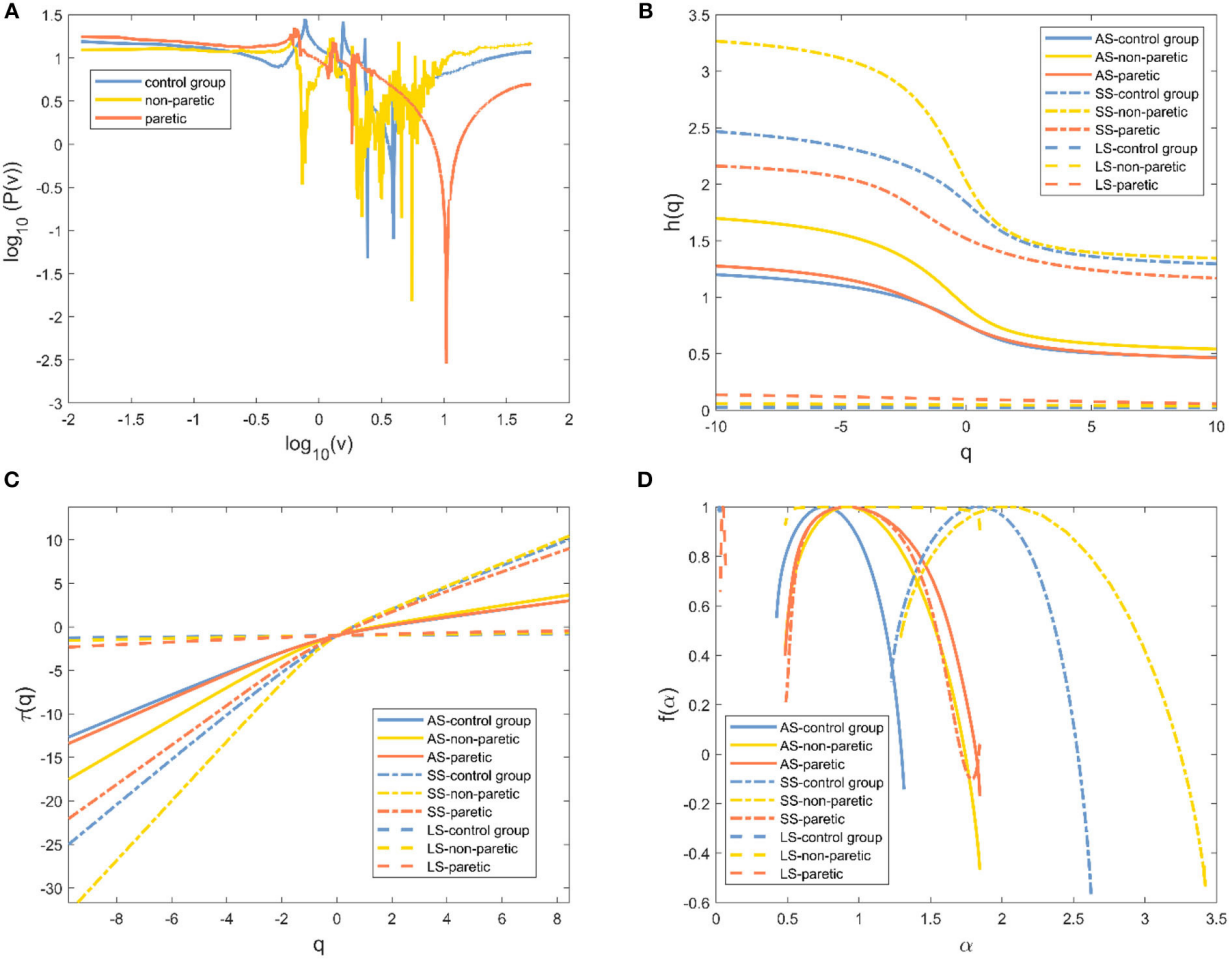
Evidence of multifractality in the knee angle time series for one of three different cases. **(A)** Fourier power spectrum of the knee angle time series. **(B)** Generalized Hurst exponent *h(q)* vs. order *q*. **(C)** Classical scaling exponent τ(*q*) vs. order *q*. **(D)** The resulting singularity spectrum *f*(α). Healthy subjects (blue line), non-paretic limbs (yellow line), and paretic limbs (red line) of a patient after stroke (AS: all-scale, —; SS: short-scale, – -; LS: long-scale, – –).

The Fourier power spectrum *P(v)* of the knee angle time series is shown in Figure 3A (*v* is the frequency, axes are shown in logarithm scale). Statistical self-similarity is manifested as a power-law scaling of the frequency distribution. The power law coefficient (slope) is changed throughout the entire v range (Figure 3A), suggesting multifractality in the knee angle time series. Multifractals reflected the different scale behavior of time series anomalies. An increase in the moment *q* could realize the transition from small anomalies to large anomalies. Thus, the representative changes of *h*(*q*) with *q* in the three cases are presented in Figure 3B. The values of *h*(*q*) decreased continuously as the moment *q* increased in Figure 3B, which indicated a multifractal behavior. The change of *h*(*q*) with *q* and the non-linear reliance of τ(*q*) on *q* reflected the multifractal characteristics of human gait in these three cases (Figure 3C). The degree of multifractality was quantitatively determined by the distribution range of fractal dimension *f*(α), which was characterized by Holder exponent α. The typical inverted parabola form could be observed in the singularity spectrum *f*(α) vs. α (Figure 3D).

### Multifractal statistics

**Figure 4:**
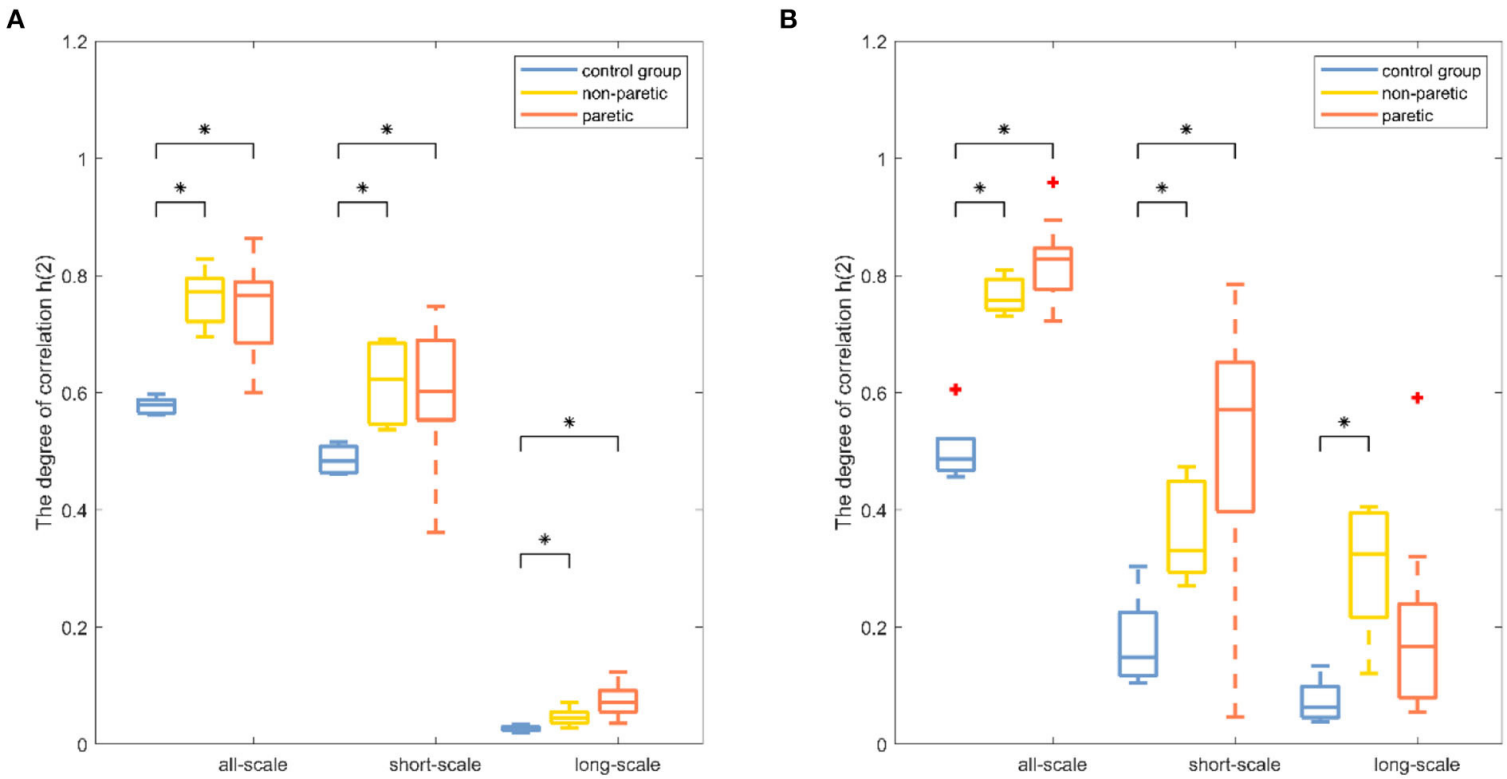
Boxplots of the fractal fluctuation results among the paretic limbs and the non-paretic limbs of a patient with stroke and the control group under three conditions (all-scale; short-scale; long-scale) during treadmill-walking tasks. Boxplots show the quartiles, the medians, and the ranges of the individual results. Outliers are indicated with + signs. * Indicates statistically significant difference at *a p*− value < 0.05.

**Figure 5:**
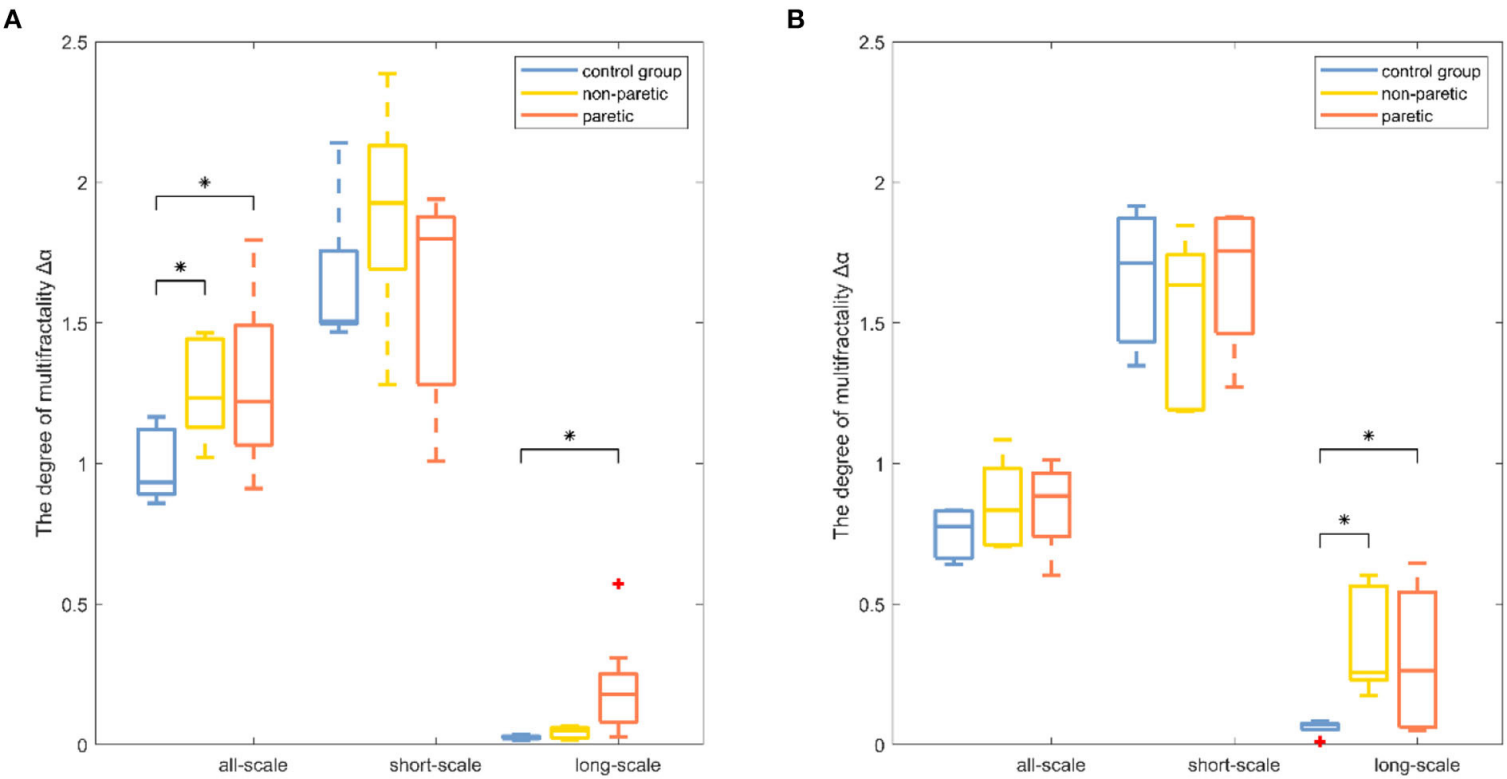
Boxplots of the degree of multifractality results among the paretic limbs and the non-paretic limbs of a patient with stroke and the control group under three conditions (all-scale; short-scale; long-scale) during treadmill-walking tasks. Boxplots show the quartiles, the medians, and the ranges of the individual results. Outliers are indicated with + signs. * Indicates statistically significant difference at *a p*− value < 0.05.

Results were reported separately for the degree of correlation and the degree of multifractality. The box plot of the multifractal analysis results showed that, when the order was *l* = 2, the time series of knee and ankle joint angles exhibited correlations and multifractality under the three fitting regions (Figures 4, 5). For the knee angles, the statistical results of *h*(2) indicated that there was persistence in all-scale and short-scale regions and anti-persistence in the long-scale region. Especially, *h*(2) corresponding to the knee angle was significantly higher in the paretic limb and the non-paretic limb of patients than in healthy subjects (*p* < 0.05). For the ankle angles, the statistical results of *h*(2) indicated that there was persistence in all-scale and anti-persistence in long-scale regions. In the short-scale region, healthy subjects and the non-paretic limb of subjects were persistence, while the paretic limb of the patients wase anti-persistence. The value of *h*(2) corresponding to the ankle angle was also significantly higher in the paretic limb and the non-paretic limb of patients with stroke than in the healthy subjects in all-scale and short-scale regions, but in the long-scale, only the non-paretic limbs of the patients were significantly higher than the healthy subjects (*p* < 0.05), although there was no significant difference in *h*(2) between the paretic and non-paretic limbs of patients after stroke. As presented in Figure 4, compared with the ankle joint angles, the knee joint angles were characterized by larger *h*(2) in the short-scale region, and the difference between the knee–ankle angles was greater in the patients. The values of Δα in knee angle were significantly higher in the paretic limb and the non-paretic limb of patients than in healthy subjects on all-scale, and the non-paretic limb of patients was higher than the healthy subjects on the short-scale region (*p* < 0.05). Although a significant difference occurred in the values of Δα in the ankle angles between the paretic limb and the non-paretic limb of patients with stroke compared with healthy subjects only in the long-scale region (*p* < 0.05), there was no significant difference on all-scale and short-scale regions.

Moreover, the corresponding random shuffle series were analyzed for all six data sets (knee and ankle angles of three groups) to determine the cause of the multifractality. As is evident in Tables 1, 2, the statistical differences of Δα and *h*(2) of each group of shuffled series were significantly smaller. The results showed that the shuffled data of the three groups all had scale exponents of about 0.5 and a multifractal strength of about 0.2. The results suggested that the multifractality in lower limb joint angles was predominantly the result of the long-range correlations.

**Table 1:** Mean and standard deviation for the shuffled series on knee angle.

**Knee**	**Control group**	**Non-paretic**	**Paretic**
All-scale Δα	0.24 ± 0.05	0.17 ± 0.06	0.19 ± 0.07
All-scale h(2)	0.50 ± 0.02	0.52 ± 0.02	0.49 ± 0. 02
Short-scale Δα	0.46 ± 0.25	0.17 ± 0.11	0.34 ± 0.17
Short-scale h(2)	0.52 ± 0.01	0.51 ± 0.01	0.50 ± 0.01
Long-scale Δα	0.42 ± 0.18	0.27 ± 0.08	0.23 ± 0.11
Long-scale h(2)	0.50 ± 0.16	0.60 ± 0.11	0.51 ± 0.09

**Table 2:** Mean and standard deviation for the shuffled series on ankle among the paretic limbs and the non-paretic limbs of a patient with stroke and the control group.

**Ankle**	**Control group**	**Non-paretic**	**Paretic**
All-scale Δα	0.10 ± 0.07	0.15 ± 0.06	0.20 ± 0.14
All-scale h(2)	0.52 ± 0.02	0.50 ± 0.03	0.48 ± 0.03
Short-scale Δα	0.16 ± 0.12	0.20 ± 0.15	0.27 ± 0.18
Short-scale h(2)	0.50 ± 0.01	0.51 ± 0.01	0.50 ± 0.01
Long-scale Δα	0.34 ± 0.12	0.55 ± 0.37	0.31 ± 0.11
Long-scale h(2)	0.56 ± 0.07	0.63 ± 0.16	0.45 ± 0.11

## Discussion

This study aimed to introduce MFDFA and two indicators, the degree of correlation and the degree of multifractality, to quantify the changes in the movement variability of the lower extremities between healthy subjects and patients after stroke. The stroke-induced destruction of the neuromuscular control system led to changes in movement variability, which manifested as multifractal and persistent increases in various scales of the knee and ankle joint angles.

The selection of proper polynomial order *l* and the scale range were two main concerns in MFDFA calculation. Three polynomial detrending functions of different orders (*l* = 1, 2, 3) were used to fit the local scale-related trends of the signals to ensure that a suitable polynomial order was used in the detrending process. As can be seen in Figure 1, the representative example of the fluctuation function *F*_*q*_ with the scale *s* was the “crossover” behavior, which might be a hallmark of postural control (21, 35). Although the slight right shift of the crossover was obvious as the DFA order *l* increased, this performance and the reported results were not sensitive to the order *l*. Therefore, *l* = 2 was selected for the remainder of this study (36). In most movement time series, crossover occurred at ~170 samples, which was assumed to be the boundary between “short-scale” and “long-scale” regions. The short-scale exponent was computed within the segment size ranging 20 < *n* < 100. Meanwhile, the long-scale exponent was evaluated in the range of 170 < *n* < 1, 000. Consistent with previous studies, the fluctuations of the joint angle signals during walking showed two typical scale regions and a crossover phenomenon, indicating that the neuromuscular system performed posture control in at least two time scales (37).

The degree of correlation, *h*(2), was an important characteristic of the walking performance because it could evaluate the movement variability during walking (15). It was interpreted as the neuromuscular control system relying on previous strides to execute future strides (18). In knee angle at all-scale and short-scale regions, *h*(2) was >0.5, indicating that the knee angle time series were persistent and long-range correlated in these scales (26). In both knee and ankle angles at the long-scale region, *h*(2) was < 0.5, indicating that these time series performed in a more anti-persistent and irregular fashion (33). The higher values of *h*(2) represented less flexibility in the neuromuscular control system that could barely adapt to the perturbations that were encountered during daily activities (15). The values of *h*(2) in the paretic limb were significantly higher than those of the healthy subjects, which was consistent with the previous study, which showed that patients after a stroke had less adaptable gait dynamics (17). It was also found that the values of *h*(2) in the paretic limb of patients with stroke were significantly higher than those of the non-paretic limb of patients with stroke, which meant that the control pattern of the paretic limb was greatly influenced by stroke (4). MFDFA provided another important indicator, the degree of multifractality Δα (21). Higher values of Δα corresponded to the more multifractal values of the timeseries dynamic interactions (29). By using wavelet-based multifractal analysis to analyze the response series of different cognitive tasks, Ihlen et al. provided quantitative support for identifying multifractality as a mathematical descriptor of dynamic interactions in human cognition (38). The multifractality in movement variability was considered by Kelty-Stephen et al. for the purpose of characterizing the dynamic interactions among the neuromuscular control system components (39). The values of Δα in the paretic limb and the non-paretic limb of patients with stroke were significantly higher in the knee angle at the short-scale region and ankle angle at the all-scale region than the corresponding values of healthy subjects in Figure 5, indicating that the patients after stroke performed a more multifractal control process (26). A more multifractal control process might result from the correctional movements in patients with stroke to maintain gait balance (40). Meanwhile, previous studies found that, when the balance feedback control (such as visual feedback) was reduced, the Δα values decreased (28). Therefore, it was suspected that patients after a stroke had a higher multifractal control process due to their increased reliance on feedback control (27). Moreover, the values of Δα corresponding to the all-scale of the patient after stroke were significantly higher than those of the healthy subjects in knee joint angles, but there was no significant difference in the ankle joint, which might reveal the different ways that the body can adjust the knee and ankle joints after a stroke (2).

The motor defects of the lower limb were usually assessed by experienced physicians or occupational therapists using clinical scales (3). At the same time, some objective evaluation methods such as kinetic, kinematics, and electrophysiological indicators were of great significance to supplement and improve traditional evaluation methods (16). In this study, MFDFA was used to study the stroke-induced movement variability changes of lower limb joint angle signals. The degree of correlation and the degree of multifractality were parameters that assessed the multifractal movement fluctuation patterns. These reflected the robust physiologic interactivity occurring within the neuromuscular control system across multiple time scales. Based on the above advantages, MFDFA of joint angle signals has the potential to be applied to the clinical evaluation of stroke-induced changes in neuromuscular control. Quantitative measurement might help therapy-based rehabilitation to optimize treatment (9). It is important to note that this study has some limitations. First, only young healthy subjects were considered. Accordingly, age-matched controls should be recruited for further study. Second, different experimental paradigms, such as joint-level variability at different speeds, might be a meaningful attempt to find the relationship between the specific control strategy and different gait patterns. Finally, in the experiment, walking performance was evaluated only through two joint angles. Future research should incorporate more evaluation indicators, such as electromyographic signals, to have a more comprehensive understanding of the mechanism of stroke-induced motor deficits.

## Data availability statement

The raw data supporting the conclusions of this article will be made available by the authors, without undue reservation.

## Ethics statement

The studies involving human participants were reviewed and approved by the Medical Ethics Committee at the Industrial Injury Rehabilitation Hospital of Guangdong. The patients/participants provided their written informed consent to participate in this study.

## Author contributions

PX and XW collected the data. PX analyzed the data and drafted the manuscript. HY and RS revised and determined the final manuscript. All authors contributed to the article and approved the submitted version.

## Funding

This work was supported in part by the National Key Research and Development Program of China under Grant 2018YFC2001600, the Guangdong Science and Technology Plan Project under Grant 2020B1212060077, the Shenzhen Science and Technology Plan Project Grant GJHZ20200731095211034, and the National Natural Science Foundation of China Grant No. 62103449.

## Conflict of interest

The authors declare that the research was conducted in the absence of any commercial or financial relationships that could be construed as a potential conflict of interest.

## Publisher's note

All claims expressed in this article are solely those of the authors and do not necessarily represent those of their affiliated organizations, or those of the publisher, the editors and the reviewers. Any product that may be evaluated in this article, or claim that may be made by its manufacturer, is not guaranteed or endorsed by the publisher.
